# Risk Factors for Infectious Adverse Events in Newly Diagnosed Acute Myeloid Leukemia Patients Treated With Venetoclax Combinations: A Retrospective Single‐Centre Real‐World Experience

**DOI:** 10.1002/cnr2.70432

**Published:** 2025-12-22

**Authors:** Olgu Erkin Çınar, Azade Kanat, Kerim Erer, Rasim Şahin, Esma Eryılmaz Eren, Esra Yıldızhan

**Affiliations:** ^1^ Department of Hematology Kayseri City Training and Education Hospital Kayseri Turkey; ^2^ Department of Infectious Diseases Kayseri City Training and Education Hospital Kayseri Turkey

**Keywords:** acute myeloid leukemia, infectious adverse events, neutropenia, venetoclax

## Abstract

**Background:**

Venetoclax‐based (ven) combinations have become a standard of care for acute myeloid leukemia (AML) patients ineligible for intensive chemotherapy. However, the associated risk of infectious adverse events (IAEs) remains a significant clinical concern.

**Aims:**

This study aimed to evaluate the incidence, characteristics, and risk factors for IAEs in newly diagnosed AML patients treated with venetoclax combinations in a real‐world setting.

**Methods and Results:**

We conducted a retrospective cohort study of AML patients treated with ven in combination with hypomethylating agents or low‐dose cytarabine (LDAC), with analyses performed on a treatment cycle basis. Clinical and laboratory data, including IAE characteristics, duration of neutropenia, and concomitant medications, were collected, and grade ≥ 2 IAEs were included according to CTCAE v5.0 criteria. The cohort included 143 treatment cycles of 43 patients, with a median neutropenia duration of 13 days (5–35). A total of 34 (23.8%) grade ≥ 2 IAEs occurred, with an incidence of 1 per 121 patient‐days. Multivariate analysis identified prolonged neutropenia (days, OR = 1.037, *p* = 0.005) and interacting concomitant medications (OR = 9.99, *p* < 0.001) as independent risk factors for IAEs. The rate of invasive fungal infections was as low as 3.5%, and the use of antifungal or antibacterial prophylaxis was not associated with a reduction in the rate of IAEs.

**Conclusion:**

IAEs remain a substantial risk in venetoclax‐treated AML patients, particularly during prolonged neutropenia and with concomitant drug interactions. Optimizing venetoclax regimens and careful management of interacting medications may mitigate these risks.

## 1 Introduction

Venetoclax (ven), a pro‐apoptotic Bcl‐2 inhibitor, is an oral agent that can be used in various combinations as the standard of care in patients with acute myeloid leukemia (AML) who are not suitable for intensive chemotherapy [1]. It is most frequently utilized in conjunction with a hypomethylating agent (HMA), such as azacitidine (aza) or decitabine (dec), or a cytotoxic agent, including low‐dose cytarabine (LDAC). Due to its high anti‐leukemic potency and limited non‐hematologic toxicity, ven is being used with increasing frequency and in an expanding spectrum of indications and clinical scenarios. Nevertheless, the most significant clinical complications are those of an infectious adverse event (IAE) [2]. Prolonged and profound neutropenias, which occur through a variety of mechanisms, particularly the severe myelotoxicity associated with ven therapy, represent a significant clinical challenge. The search for the ideal approach for IAE risk minimization and optimal management in AML patients treated with ven combinations continues, including shortening the duration of ven therapy, but IAE‐focused well‐established studies are lacking [3]. The aim of this study was to identify IAEs and associated complications such as length of hospitalization, grade and duration of neutropenia in newly diagnosed AML patients treated with ven combinations in a real‐world setting.

## 2 Patients and Methods

### 2.1 Sample and Data Collection

This is a single‐center retrospective cohort study conducted at Kayseri City Training and Research Hospital, a tertiary regional reference center in Turkey. Patients to be included in the sample were retrospectively screened from the electronic database of the hospital. Newly diagnosed AML patients aged ≥ 18 years and treated with first‐line ven combinations (ven‐HMA, ven‐aza, and ven‐dec, or ven‐LDAC) who were admitted between July 1, 2018, and July 1, 2024, were included in the study. Patients with active grade ≥ 3 bacterial or invasive fungal infections (IFI) before the initiation of remission induction course and those with a follow‐up period of less than 4 weeks after the start of therapy were excluded from the study. Analyses were conducted using pairwise deletion for missing data, meaning that individual patients contributed to each statistical test only if data for the specific variable of interest were available but remained included in other analyses involving variables with complete data.

After the AML cohort was established, basic demographic data, dates of diagnosis, each treatment cycle, and last visits; blood count data, concomitant medications, clinical, radiological, or laboratory data regarding IAEs, infection sites, and IAE grades were extracted from records.

### 2.2 The Assessment of Therapy and Adverse Events

Follow‐up began on the date of ven initiation and continued until death, treatment discontinuation, or last available medical record within the study period. The ven combinations used by the patients (ven‐aza, ven‐dec, or ven‐LDAC), the dose and duration of the ven used, other medications, and presence of drug interaction were recorded. Adverse events were graded according to the Common Terminology Criteria for Adverse Events (CTCAE) v5.0 [4]. As the primary factor influencing the incidence of IAEs associated with venetoclax is the duration of treatment, each treatment cycle was meticulously documented and a “IAEs per 100 patient‐day” and rate metrics were employed. To illustrate, if four distinct IAEs were identified in three patients over a total of 200 days of venetoclax use, the rate was calculated as 2 per 100 patient‐days. The duration of neutropenia, drug interactions, and prophylactic anti‐infective treatments were recorded. The duration of neutropenia was defined as the number of days from the last day of ven dose in each cycle until the absolute neutrophil count (ANC) reached ≥ 500/μL. In the event of any IAE being identified (either bacterial or IFI), the specific site affected and severity were recorded.

### 2.3 Statistical Analysis

The statistical analysis was performed using the IBM SPSS Statistics for MacOS, Version 27.0 (IBM Corp, Armonk, NY). Descriptive statistics of the sample were presented in terms of frequencies (%), means (standard deviation, SD), and medians (interquartile range, IQR), depending on the type and distribution of the variables. The normality of the data distribution was assessed using the Shapiro–Wilk test. Categorical variables were compared using the chi‐square test (or Fisher's exact test, if the expected frequency in any value was < 5). Differences in the means of parametric continuous variables between two independent groups were assessed using the independent‐samples *t*‐test. For non‐parametric continuous variables, median values between two groups were examined using the Mann–Whitney *U* test, whereas the Kruskal–Wallis test was employed for three or more groups. A logistic regression model was constructed to evaluate the impact of multiple independent variables on the categorical dependent variable. Statistical significance was determined at a *p* value < 0.05. Survival analyses were performed using the Kaplan–Meier method, and differences between patient groups were assessed with the log‐rank test.

## 3 Results

The study sample comprised a total of 43 patients (10 females, 23%) that met the study criteria and received 143 treatment cycles. The median age at diagnosis was 70 (58–71) years. Patients received a median of 4 (3–6) treatment cycles. The median ven dose was 400 (200–400) mg/day. Regarding combination regimens, 24 patients (56%) received ven‐aza, 10 (23%) received ven‐dec, and 9 (21%) were treated with ven‐LDAC. The median follow‐up duration was 8.4 (3.8–15.9) months, and a total of 4098 venetoclax patient‐days were recorded.

While following the first treatment cycle, the median duration of neutropenia was 33 days (12–42); among all cycles, the median duration of neutropenia was 13 days (5–35). Granulocyte colony‐stimulating factor (G‐CSF) support, exclusively with filgrastim, was administered in 14 out of 143 treatment cycles (9.8%). There was no statistically significant difference in neutropenia duration between cycles with and without G‐CSF support (16.5 [11.5–58] vs. 12 [5–39.5] days, respectively; *p* = 0.81). Throughout 143 treatment cycles, accounting for 4098 patient‐days of follow‐up, a total of 34 grade ≥ 2 IAEs occurred. Under ven therapy, the rate of grade ≥ 2 IAEs was found to be 0.83 per 100 patient‐days (0.83%) and 24 per 100 patient‐cycles (24% for each cycle). The distribution of these events according to grade, site of infection, and use of antimicrobial prophylaxis is presented in Table 1.

**TABLE 1:** Summary of infectious adverse events, prophylaxis, and severity (*n* = 143 cycles).

Variable	*n* (%)
Antibacterial prophylaxis	17 (11.9)
Trimethoprim/sulfamethoxazole	11 (64.7)*
Ciprofloxacin	4 (23.5)*
Levofloxacin	3 (17.6)*
Antifungal prophylaxis	11 (7.7)
Posaconazole	3 (27.3)**
Fluconazole	8 (72.7)**
Primary infection sites^a^	
Pulmonary	9
Urinary system	6
Without an identifiable source	6
Skin or soft tissue	4
Gastrointestinal	3
Catheter site	3
Sinuses	1
Abdominal compartment	1
Bacteremia (isolated or complicating)	15
Infection grades^b^	
Grade 2	10 (33.3)
Grade 3	9 (30.0)
Grade 4	5 (16.7)
Grade 5	6 (20.0)
Invasive fungal infections	5
Pulmonary involvement	4
Sinus involvement	1
Grade 3	2 (40.0)
Grade 4	2 (40.0)
Grade 5	1 (20.0)
Voriconazole therapy	3
Amphotericin B therapy	2

*Note:* *, ** denote percentages for prophylaxis subcategories are shown relative to the total number of cycles that specific prophylaxis type given.

Abbreviations: IAE, infectious adverse event; IFI, invasive fungal infection.

^a^ Primary infection sites listed are based on 34 IAEs in total (23.8% of 143 cycles). Some patients may have had more than one infection site.

^b^ Thirty cases had documented infection grades (sum of Grades 2, 3, 4, and 5 = 30).

Among 17 treatment cycles with antibacterial prophylaxis, 6 bacterial IAEs developed (35%), whereas 28 IAEs developed among 126 cycles without prophylaxis (22%). No statistically significant difference was observed between the groups (*p* = 0.185). Among 10 treatment cycles with antifungal prophylaxis, no IFIs were observed. Five IFIs occurred during 133 cycles (3.76%) without prophylaxis. This difference in groups did not show statistical significance (*p* = 0.668).

Across a total of 33 treatment cycles with ven, concomitant medications leading to 9 minor (4 ciprofloxacin, 5 others) and 24 major drug interactions (1 carbamazepine, 2 clarithromycin, 3 posaconazole, 8 fluconazole, and 10 voriconazole) were identified. In 5 of the 24 major episodes, no ven dose modification was documented. Ven doses were modified in 11 of the 45 patients due to drug interactions. Grade ≥ 2 IAEs occurred in 7 of 11 patients (63.6%) with dose modification, and in 22 of 34 patients (64.7%) without dose modification. There was no statistically significant difference between the two groups (*p* = 1).

In multivariate logistic regression analysis, after adjusting for age, ven dose, interacting concomitant drug use, antimicrobial prophylaxis use, and duration of neutropenia (days), the interacting concomitant drug use was significantly associated with a higher risk of IAEs (OR = 9.99, 95% CI [3.176–31.447], *p* < 0.001). Similarly, longer duration of neutropenia was independently associated with an increased risk of IAEs (OR = 1.037, 95% CI [1.011–1.064], *p* = 0.005). Patients who developed bacterial grade ≥ 2 IAEs had a significantly longer median duration of neutropenia (28.5 [12–51.5] days) compared to those who did not (7 [5–25.5] days). This difference was statistically highly significant (*p* < 0.001). The median duration of neutropenia was 7 (5–32) days in cycles without interacting concomitant drug use, compared to 23 (6–36) days in cycles with major drug interaction, and this difference was statistically significant (*p* = 0.034). Analyses based on median duration of neutropenia revealed no significant differences between groups according to IAE grade (*p* = 0.842), IFI development (*p* = 0.593), or median ven dose (*p* = 0.368).

**FIGURE 1:**
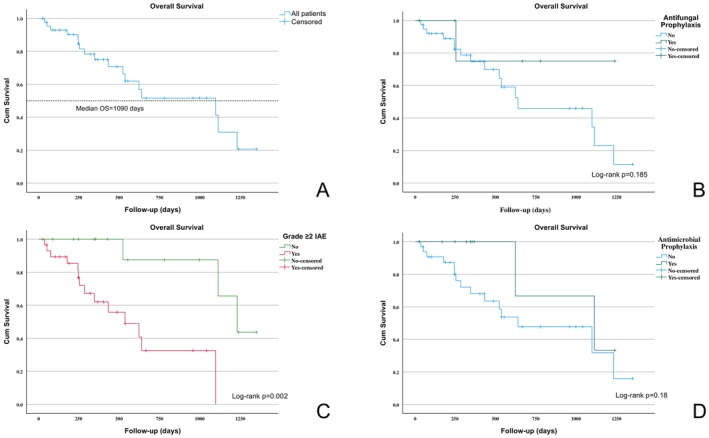
Kaplan–Meier overall survival (OS) curves. (A) OS in the entire cohort. (B) OS according to the use of antifungal prophylaxis. (C) OS according to the presence or absence of grade ≥ 2 infective adverse events. (D) OS according to the use of antimicrobial prophylaxis (antiviral, antibacterial, or antifungal).

The median overall survival (OS) of the cohort was 1090 days. Kaplan–Meier curves illustrating OS for the entire cohort, as well as subgroup analyses based on the occurrence of infective adverse events (IAEs), antimicrobial prophylaxis, and antifungal prophylaxis, together with log‐rank test comparisons, are presented in Figure 1.

## 4 Discussion

Ven‐based combinations have changed the treatment landscape of the patients who are not suitable for intensive chemotherapy and extended the survival compared to other classical non‐intensive regimens [5, 6, 7]. However, ven‐based therapies significantly increase the risk of high‐grade febrile neutropenia. In one meta‐analysis conducted a combined analysis of randomized controlled trials (RCT), 38.4% of patients experienced high‐grade febrile neutropenia compared to 22.2% in the control group, with a relative risk (RR) of 1.73 [8]. The relatively lower incidence of grade ≥ 2 IAEs in our cohort, comparable to that reported in the control arms of RCTs, may be attributed to several factors. These include the inclusion of generally older patients in RCTs than in the real‐world setting, the optimization of ven dose and duration modifications over time, and the more rigorous recording of IAEs within the setting of clinical trials, particularly compared to retrospective analyses. In a real‐world study conducted in China, the median duration of neutropenia in newly diagnosed AML patients treated with ven‐aza was reported to be 16 days [9]. This duration is quite comparable to the results of our study and highlights the high risk of infection. Moreover, neutropenia during the induction period is expected to last longer. Since the duration of neutropenia is one of the most critical factors determining the risk of IAEs, it raises the question of whether shortening the duration of ven could reduce both neutropenia and IAEs. This topic has been the focus of numerous studies. For instance, according to the results of a phase II study, a 14‐day schedule of ven‐aza may reduce toxicities while maintaining comparable response rates in newly diagnosed acute myeloid leukemia patients [10]. In our study, we confirmed that the duration of neutropenia significantly increases the risk of IAEs, as expected, and this finding is consistent with data reliability. Considering the available literature regarding the use of G‐CSF as an alternative risk‐reducing measure, it is noteworthy that DiNardo et al. demonstrated that the use of G‐CSF did not significantly reduce the duration of neutropenia in an analysis evaluating results from phase 3 trials [11]. Therefore, it can be suggested that routine use of G‐CSF may not sufficiently decrease the risk of IAEs.

In a retrospective cohort study conducted by On et al., the incidence of IFIs in AML patients treated with venetoclax is 5.1%, and the incidence did not differ significantly according to age, antifungal prophylaxis use, or disease status [12]. Similar to the incidence observed in our study, the authors demonstrated an overall low risk of developing IFI with the use of ven‐HMA. Furthermore, high‐impact studies indicate that antifungal prophylaxis, due to its potential for added toxicity, may negatively impact patient survival [13]. In light of these numerous similar findings, routine antifungal prophylaxis may not be mandatory and should be considered in an individual and local risk‐based approach, given both the low IFI incidence and reports of reduced survival associated with prophylaxis. The low rate of IFI and the limited use of antifungal prophylaxis observed in our cohort should be interpreted in the context of aforementioned data and our institutional policies, and therefore may not be generalizable to all populations.

We also demonstrate a statistically highly significant increased IAE risk for treatment cycles where a drug known to interact with ven was used, including but not limited to antifungals. Our observation further supports the potential negative consequences of antifungal use described in the literature, such as prolonged neutropenia and even decreased survival [13, 14]. Furthermore, interactions between non‐azole anti‐infectives, such as clarithromycin, and agents less commonly used by hematologists, such as carbamazepine, may be overlooked, as observed in our cohort, or optimal dose modifications may not be well established, potentially leading to unresolved risks. For instance, while the effect of ciprofloxacin on CYP3A4 varies in the literature from negligible to moderate inhibition, the manufacturer's ven drug interaction brochure recommends a 50% dose reduction with concomitant use [15]. Notably, we observed no venetoclax dose modifications during any treatment cycles involving concomitant ciprofloxacin use in our cohort. This underscores the need for increased awareness regarding the extensive drug interaction profile of ven.

Strengths of our study include its real‐world setting, reflecting everyday clinical practice, and the provision of detailed descriptive findings on infectious complications and drug interactions. These represent clinically relevant potential risks that necessitate careful monitoring in clinical practice. Nevertheless, our study has several important limitations. Most notably, it is a retrospective cohort study and thus susceptible to bias. Among potential sources of bias, patient selection criteria at our center for those deemed “unfit for intensive therapy” may not precisely align with criteria employed in RCTs; consequently, our cohort may include relatively fit or younger, “grey zone” patients who inherently possess a lower risk of infectious adverse events. Other significant limitations include the single‐center design and the relatively small sample size. Institutional policies, particularly regarding antimicrobial prophylaxis choices, substantially influence clinical practice; therefore, our findings may not be generalizable to other centers. Additionally, the limited sample size precluded comprehensive subgroup analyses, such as those based on AML subtype or comorbidity categories. Furthermore, although this study focused directly on ven treatment and associated IAEs, the analyses did not incorporate other potential factors such as disease subtype, remission status, and comorbidities, primarily due to sample size limitations.

## 5 Conclusion

In conclusion, antimicrobial prophylaxis may not be universally required for all patients; the necessity for concurrent azole use with ven should be evaluated on an individual basis, and minimizing the duration of neutropenia may be a key risk reduction goal associated with the use of ven combinations in AML patients, including shortening the duration of ven exposure after remission and avoiding drug interactions unless absolutely necessary. However, numerous confounding factors, including AML subtype and remission status, comorbidities, additional agents, and healthcare‐associated interventions, warrant focused further well‐established prospective studies to elucidate optimal management.

## Author Contributions

**Olgu Erkin Çınar:** conceptualization, writing – original draft, methodology, formal analysis, investigation, software. **Azade Kanat:** writing – review and editing, data curation. **Kerim Erer:** data curation, writing – review and editing. **Rasim Şahin:** writing – review and editing, data curation. **Esma Eryılmaz Eren:** writing – review and editing, supervision. **Esra Yıldızhan:** supervision, writing – review and editing, conceptualization.

## Funding

The authors have nothing to report.

## Ethics Statement

This study was conducted in accordance with the principles of the Declaration of Helsinki. Ethical approval was obtained from the Kayseri City Hospital Non‐Interventional Clinical Research Ethics Committee (approval number: 184, date: September 16, 2024). Patients provide general consent for their clinical, laboratory, and radiological data to be used in anonymized retrospective research when they apply to our center. This study has a retrospective design and was conducted using existing data. Therefore, in accordance with national ethical and legal regulations, obtaining informed consent from patients was not required.

## Conflicts of Interest

The authors declare no conflicts of interest.

## Data Availability

The data that support the findings of this study are available on request from the corresponding author. The data are not publicly available due to privacy or ethical restrictions.
